# Hepatitis D Virus and Hepatocellular Carcinoma

**DOI:** 10.3390/v13050830

**Published:** 2021-05-04

**Authors:** Patrizia Farci, Grazia Anna Niro, Fausto Zamboni, Giacomo Diaz

**Affiliations:** 1 Hepatic Pathogenesis Section, Laboratory of Infectious Diseases, National Institute of Allergy and Infectious Diseases, National Institutes of Health, Bethesda, MD 20892, USA; 2 Gastroenterology Unit, Fondazione IRCCS “Casa Sollievo Sofferenza”, San Giovanni Rotondo, 71013 Foggia, Italy; g.niro@operapadrepio.it; 3 Liver Transplantation Center, Azienda Ospedaliera Brotzu, 09134 Cagliari, Italy; faustozamboni@yahoo.it; 4 Dipartimento di Scienze Biomediche, Università di Cagliari, 09124 Cagliari, Italy; gdiaz@unica.it

**Keywords:** Hepatitis D virus, cirrhosis, hepatocellular carcinoma, HBV replication, HDV replication, transcriptomics

## Abstract

Hepatitis D virus (HDV) is a small, defective RNA virus that depends on hepatitis B virus (HBV) for virion assembly and transmission. It replicates within the nucleus of hepatocytes and interacts with several cellular proteins. Chronic hepatitis D is a severe and progressive disease, leading to cirrhosis in up to 80% of cases. A high proportion of patients die of liver decompensation or hepatocellular carcinoma (HCC), but the lack of large prospective studies has made it difficult to precisely define the rate of these long-term complications. In particular, the question of whether HDV is an oncogenic virus has been a matter of debate. Studies conducted over the past decade provided evidence that HDV is associated with a significantly higher risk of developing HCC compared to HBV monoinfection. However, the mechanisms whereby HDV promotes liver cancer remain elusive. Recent data have demonstrated that the molecular profile of HCC-HDV is unique and distinct from that of HBV-HCC, with an enrichment of upregulated genes involved in cell-cycle/DNA replication, and DNA damage and repair, which point to genome instability as an important mechanism of HDV hepatocarcinogenesis. These data suggest that HBV and HDV promote carcinogenesis by distinct molecular mechanisms despite the obligatory dependence of HDV on HBV.

## 1. Introduction

HCC is the fifth most common human cancer and the third leading cause of cancer-related death worldwide [1]. It is one of the most heterogeneous cancers [2], and the lack of markers for the early diagnosis as well as the lack of effective treatment strategies have made it one of the most challenging diseases. In contrast to the decreased mortality observed over the past decades for several tumors, liver and bile duct cancers continue to show dramatic increases in their rates of death [3]. HCC usually occurs in the setting of underlying liver disease, with cirrhosis being present in 80–90% of cases. Hepatitis B virus (HBV) and hepatitis C virus (HCV) account for more than 70% of all HCC globally [4]. However, the role of hepatitis D virus (HDV) in HCC and whether HDV is an oncogenic virus remain to be established. One of the most important confounding factors in dissecting the role of HDV in hepatocarcinogenesis is the unusual nature of HDV, a hybrid virus that incorporates the hepatitis B virus surface antigen (HBsAg) as its envelope protein [5]. As a consequence, HDV can establish infection only in individuals who simultaneously acquire HBV or by superinfection of a chronic HBsAg carrier [6]. It is estimated that 15–20 million HBsAg carriers are coinfected with HDV worldwide [7], although recent studies have suggested either a higher number, between 42 and 72 million [8,9], or a lower number of around 12 million [10]. These different estimates reflect the wide heterogeneity in the recruitment of patients, as well as differences in methodologies used for the diagnosis of HDV infection [11]. As a consequence, the true prevalence of HDV infection worldwide still remains unknown. In this review, we will discuss the uniqueness of HDV, the risk of developing HCC in the natural history of chronic hepatitis D compared to HBV monoinfection, and the potential molecular mechanisms of HDV hepatocarcinogenesis.

## 2. Uniqueness of Hepatitis D Virus

To understand the pathogenesis of the disease caused by HDV, it is important to highlight the uniqueness of this virus, one of the most interesting and unusual human pathogens, discovered by Rizzetto et al. in Italy more than 40 years ago [12]. HDV is a satellite virus of HBV since it is coated by HBsAg. This implies that HDV can infect only individuals who are simultaneously infected by HBV [13]. The virus is a 36 nm particle, containing, in its interior, the smallest known genome in animal virology, a single-stranded circular RNA genome of about 1.7 KB. HDV contains a nucleocapsid, a ribonucleoprotein complex formed by the RNA genome with the hepatitis delta antigen (HDAg) [13,14]. The genome encodes a single structural protein, the HDAg, which exists in two isoforms, the small HDAg (S-HDAg; 195 amino acids) and the large HDAg (HDAg-L; 214 amino acids). The L-HDAg contains an isoprenylation motif at the C-terminus that is essential for anchoring the HDV ribonucleoprotein to the HBsAg during virion assembly. The S-HDAg is essential for viral replication, whereas L-HDAg is required for virion assembly and inhibits viral replication. The most peculiar feature of HDV is the replication of the viral genome, which occurs in the nucleus of hepatocytes as the liver is the only organ in which HDV replicates [14]. Unlike other RNA viruses, HDV does not encode its own polymerase but uses the host RNA polymerase II for replication, which normally copies double-stranded DNA templates [13]. HDV has the unique ability to redirect this cellular enzyme to transcribe the HDV RNA genome [14]. However, although HDV is a satellite virus of HBV, the replication of HDV is completely autonomous from that of HBV [14]. HBsAg is critical for virion assembly and release, and the propagation of HDV to infect other hepatocytes, and is the only HBV contribution to the life cycle of HDV [13].

## 3. Risk of Developing HCC in Patients with Chronic HBV-HDV Infection and Comparison with Chronic HBV Monoinfection

Over the past two decades, universal HBV immunization, improved socioeconomic conditions and educational campaigns against HIV infection have led to a significant decline in the incidence of HDV infection in developed countries [15]. This decline was associated with a dramatic decrease in fresh forms of hepatitis D in parallel to an increase in the proportion of cirrhosis in patients infected decades ago [16]. Chronic hepatitis D is a severe and rapidly progressive liver disease. However, the course of the disease is variable and heterogeneous in different regions of the world [17]. Chronicity develops in over 90% of HBV-superinfected individuals, and cirrhosis is reported in 70% to 80% of the cases within 5 to 10 years after infection [18]. Once established, cirrhosis may remain a stable disease for many years, although a high proportion of patients develop long-term complications [19]. However, the lack of large prospective studies has made it difficult to evaluate the rate of long-term complications during the natural history of chronic hepatitis D, i.e., hepatic decompensation and HCC, the most important causes of liver-related death or liver transplantation. Thus, the outcome of HDV disease and related complications has generally been inferred by retrospective studies of long-term follow-up or in cross-sectional studies. In an Italian study, a total of 188 patients, mainly with cirrhosis at presentation, were observed for a mean period of 7.8 years. For the entire cohort, the median probability of survival free of major events, either decompensation or HCC, from the initial diagnosis, was 28 years [20]. The number of complications was determined by the underlying stage of disease with a significant difference at 10 years between patients with cirrhosis (41% complications) and those with chronic hepatitis (18% complications). The estimated annual incidence of liver decompensation in HDV cirrhosis has been estimated to be between 2.6% and 3.6%, and that of HCC between 2.6% and 2.8% [20,21,22]. In other reports, in which the population was mostly represented by native Europeans, complications related to portal hypertension were observed more frequently than the development of HCC [22,23,24,25]. In one study from Germany, hepatic decompensation was shown to be the primary cause of mortality [26].

However, in these studies, the rate of HCC development was evaluated only in HBV-HDV coinfected patients and was not compared with that of HBV monoinfection. Thus, these data are confounded by the hybrid nature of HDV, and, in these patients, it is difficult to dissect the relative contribution of the two viruses, HBV and HDV, to HCC pathogenesis. Indeed, different estimates were obtained when the risk of HCC development was evaluated in HDV-infected patients compared with those with HBV monoinfection. Fattovich et al. showed a risk of HCC that was 3-fold higher in HDV cirrhosis than in HBV cirrhosis [21]. In Mongolia, where the prevalence of HDV is one of the highest in the world [8], HBV-HDV coinfection was strongly associated with HCC development, which occurred at younger age [27]. The risk was also confirmed in a population-based study, in which the Swedish Hospital Discharge Register and Outpatient Registry was used to identify 9160 patients with chronic HBV infection between 1997 and 1998. Among them, 650 individuals were HDV infected with an acute (323 subjects) or chronic (327 subjects) form of HDV disease. The risk of HCC in HDV-infected patients was 6-fold higher than that observed in HBV [28], indicating that HDV is a strong risk factor for HCC. The risk was also confirmed in another large nationwide retrospective study of all veterans who tested positive for HBsAg in the United States between 1999 and 2013. Out of 25,603 HBsA-positive veterans, only 2175 (8.5%) were tested and 73 (3.4%) were positive for HDV [29]. However, most of them (59%) were also coinfected with HCV. HDV was commonly linked to substance abuse disorders and cirrhosis. The incidence rate of HCC was 2.9-fold higher in HDV-positive than that in HDV-negative individuals. The association was also independent of the presence of cirrhosis, suggesting that HDV may be directly oncogenic.

The interaction of HDV with HIV-1 leads to a more rapid liver fibrosis progression. In a nationwide Swiss HIV Cohort Study, HDV-infected patients were more likely to be intravenous drug users (60.6% vs. 9.1%) and to have a positive HCV serology (73.1% vs. 17.8%) compared to HDV-uninfected individuals. HDV infection was strongly associated with overall death (adjusted hazard ratio 2.33, 95% CI 1.41–3.84), liver-related death (7.71, 3.13–18.97), and the occurrence of HCC (9.30, 3.03–28.61) [30].

This risk was further confirmed in a recent extensive study by Alfaiate et al. [31], who conducted the first meta-analysis of a large series of observational studies, based on 93 studies that comprised 68 case-control studies and 25 cohort studies, to assess the association between chronic hepatitis D and HCC [31]. The results of this meta-analysis, despite the heterogeneity of the studies, confirmed that patients with HDV had a significantly higher risk of developing HCC than those with HBV monoinfection. This increase was even more evident when the analysis was restricted to studies performed after 2010, which were more robust and better designed. Another important factor to consider is the improvement of cirrhosis care in developed countries, which may have favored the emergence of long-term complications such as HCC, which were less evident in the early years after the discovery of the virus [31]. This study also suggests that although most HDV-infected patients are also coinfected with HCV, which is a well-known risk factor for HCC, an increased risk of HCC in HDV-infected individuals was confirmed in 24 studies after the exclusion of HCV-infected patients [31]. A higher increase in HCC development was also observed in patients coinfected with HDV and HIV-1, where HDV appears to be a major driver of HCC, probably due to the underlying immunodeficiency [31].

Abbas et al. attempted to identify differences at presentation between HDV- and HBV-associated HCC by comparing clinical features and tumor characteristics [32]. They found that HDV-HCC was associated more frequently with a decreased liver size, indirect evidence of a more severe portal hypertension and lower platelet counts compared to HBV-HCC, which instead was more commonly associated with multifocal tumors and high levels of alpha-fetoprotein [32]. Although a large number of consecutive HCC patients were included in this report (92 patients seropositive for antibody against HDV and 92 HBsAg positive and anti-HDV negative), a limitation of this study was the fact that these patients were identified in a tertiary care hospital among those hospitalized for advanced liver disease and were not derived from a surveillance program [32].

## 4. HDV and Carcinogenesis

Although a recent comprehensive meta-analysis study provided evidence that chronic hepatitis D is associated with an increased risk of developing HCC, compared to HBV monoinfection [31], there are very limited data on the molecular mechanisms whereby HDV promotes liver cancer. Due to the vital dependence of HDV on HBV, it is unknown whether HCC is the result of a cumulative effect of both HBV and HDV, an effect of the underlying cirrhosis, or a direct oncogenic effect of HDV. The introduction of genomics has provided important tools for dissecting the pathogenesis of complex diseases in which thousands of genes are simultaneously dysregulated. Several studies have defined the molecular profiles of HBV-and-HCV-associated HCC [33,34,35,36]. In contrast, there is limited information on the genomic signature of HDV or on the levels of HDV replication within the tumor. Some of the major obstacles in defining the role of HDV in HCC pathogenesis include the limitations of available experimental systems, and the difficulties in obtaining liver samples from patients with HDV-associated HCC, in particular paired liver specimens from the tumor and surrounding nontumorous tissue.

## 5. Levels of HDV and HBV Replication in HCC-Associated with HDV

The replicative activity of HDV and HBV is generally predictive of a poor prognosis. In a study by Romeo et al. [22], 46 out of 146 cirrhotic patients developed HCC over a mean period of 7 years since the diagnosis of cirrhosis. There was no significant difference in the levels of HDV viremia between patients who developed HCC and patients with cirrhosis who did not develop long-term complications. High levels of HDV viremia in non-cirrhotic patients were independently associated with progression to cirrhosis and development of HCC, while, once cirrhosis was established, the role of HDV replication as a predictor of a negative outcome decreased [37]. In a large multi-ethnic cohort study of HDV-infected patients in France, with immigrants being the most represented population, persistent HDV replication was associated with a significantly increased risk of all liver complications, and with HCC occurrence [38]. However, in a single-center cohort study of anti-HDAg-positive patients [39], HCC only occurred in the presence of liver cirrhosis, whereas HDV RNA positivity at baseline did not correlate with any clinical outcome. This study highlighted the risk of developing HCC also in patients who are HDV- RNA-negative and have advanced liver fibrosis [39].

Although a correlation between the levels of HDV replication and the risk of liver complications has been documented [20,40], there is limited information on the levels of HDV and HBV replication in patients with HDV-associated HCC. Even more limited are the data on the levels of HDV replication within the tumor and in the surrounding nontumorous tissue. Thus, it is difficult to infer the levels of HDV replication within the tumor from studies conducted almost exclusively on serum samples. In a previous study performed by our group in HCV-HCC, a significant drop in HCV RNA was exclusively associated with malignant hepatocytes, whereas the levels of HCV replication in serum were similar between cirrhotic patients with HCC and those with non-HCC [41]. Recently, Diaz et al. [42] investigated the levels of HDV replication within the tumor by analyzing paired tumor and nontumorous tissue of five patients with HDV-associated HCC, and the results were compared with the levels in HDV cirrhosis without HCC in patients who underwent liver transplantation for end-stage liver cirrhosis. Although the number of patients included in this study was limited, these patients were well characterized, all were Caucasian, and they were followed in a single Italian center. In patients with HDV-HCC, there was a sharp decline in HDV RNA within the tumor in two of the five patients studied, in whom up to 17 liver specimens were examined. The drop in HDV RNA occurred between the periphery of the tumor and the perilesional area, whereas the levels were similar in all the surrounding nontumorous areas. In the remaining three patients, the levels between tumor and nontumorous tissue were similar. Overall, the levels of HDV RNA tended to be lower in the tumor than in the surrounding nontumorous areas. Thus, this study demonstrated that the pattern of HDV replication within the tumor was not the same in all cases, and that the restricted viral replication within the tumor would have been missed by testing only the levels of HDV viremia, which were similar between HDV-HCC and non-HCC cirrhosis. By immunohistochemistry, HDAg was detectable in only three of five tumors, but in all five nontumorous tissue samples as well as in all non-HCC HDV cirrhosis, further confirming that hepatitis viruses do not replicate well in malignant hepatocytes. In addition, the number of nuclei of malignant hepatocytes positive for HDAg was remarkably lower in the tumor than in paired nontumorous tissue. Additional studies are necessary to investigate whether malignant hepatocytes lack expression of host factors that are important for entry or viral replication. Recently, we demonstrated that the restricted HCV RNA replication within the tumor [41] was associated with the downregulation of tumor-associated calcium signal transducer 2 (TACSTD2), the second most downregulated gene in primary HCV-HCC tissue, which is essential for maintaining the proper cellular localization of CLDN1 and OCLN, two major HCV-entry cofactors [43]. TACSTD2 gene silencing significantly inhibited HCV infection with a pan-genotype effect that occurred at the level of viral entry, indicating that TACSTD2 is a novel host cofactor for HCV entry [43].

Although HDV is a defective virus that always coexists with HBV, knowledge on the levels of HBV DNA within the tumor is limited to a single study [42]. By analyzing the intrahepatic HBV DNA in paired tumor and nontumorous tissue, Diaz et al. showed [42] that HBV DNA levels were extremely low or undetectable both in the serum and in the liver of all patients with HDV-HCC, as well as in non-HCC HDV cirrhotic livers, consistent with the low levels of HBV replication, typically associated with chronic HDV disease [6]. Accordingly, HBcAg was undetectable in any tumor and nontumorous tissues, as well as in all but one of seven patients with non-HCC HDV cirrhosis [42]. HBV replication was lower in HCC and in non-HCC HDV cirrhosis compared to those detected in patients with HBV monoinfection associated with HCC, although these patients were all anti-HBe positive and under therapy with nucleos(t)ide analogues prior to surgery [36]. Thus, these data indicate that HCC-HDV is not associated with an increase in HBV replication.

## 6. Potential Oncogenic Mechanisms of HDV: In Vitro Studies

Chronic viral hepatitis is associated with inflammation, oxidative stress and DNA damage, leading to liver fibrosis progression and, ultimately, to cirrhosis and HCC. Cirrhosis is the most important risk factor for the development of HCC, although HCC can also arise in a non-cirrhotic liver [44]. Hepatocarcinogenesis has been associated with complex alterations of molecular signaling pathways [45]. However, there are very limited data on the potential oncogenic mechanisms of HDV. By proteomic analysis in HEK-293 cells, Mendes et al. demonstrated that 89 proteins were differentially expressed as a result of HDV replication [46]. Using a system biology approach, they found that the G2/M DNA damage checkpoint and pyruvate metabolism were among the most affected pathways, with cancer as the disease more closely associated with HDV replication [46], suggesting that these mechanisms may play a role in HDV hepatocarcinogenesis.

Several molecular pathways could potentially be implicated in HDV HCC (reviewed in [47,48]). Transforming growth factor-β (TGF-β) modulates various cellular processes, including proliferation, differentiation, wound repair, and apoptosis [49]. The TGF-β pathway is a major modulator of hepatic fibrosis by stimulating the transcription of fibrosis-associated genes [50], but a functional role has been also reported in hepatocarcinogenesis [49]. Choi et al. demonstrated that L-HDAg, but not S-HDAg, activates the TGF-β and c-Jun signaling cascades. These effects are mediated by isoprenylation of L-HDAg [51]. Other in vitro studies have shown that L-HDAg, but not S-HDAg, regulates several cellular functions by activating nuclear factor kappa B (NF-kB) via the induction of tumor necrosis factor-α (TNF-α) [52] and the signal transducer and activator of transcription-3 (STAT-3) [53]. Using a hepatoma cell line (Huh7), Williams et al. [54] demonstrated that L-HDAg induces oxidative stress through the activation of Nox4 gene expression, which, in turn, promotes an increase in the release of reactive oxygen species (ROS). These activate STAT-3 and NF-kB, both of which are implicated in several cellular signaling pathways involved in inflammation, apoptosis, cell proliferation, and tumor development. In the presence of antioxidants or calcium inhibitors, this activation was dramatically reduced [54]. Several studies indicate that human cancers contain constitutively activated STAT-3 protein [55], which is also persistently active and mediates cellular transformation in many immortalized cell lines, acting as an oncogene [56]. Thus, the activation of STAT-3 could be another mechanism whereby HDV promotes carcinogenesis.

Goto et al. [57] investigated the role of the two isoforms of the HDAg, but they found that only the large isoform, L-HDAg, activates serum response factor-associated transcription (SRF), which was previously reported to mediate serum- and growth factor-induced transcription of the c-fos proto-oncogene [58,59]. Subsequently, the same group demonstrated that HBV X protein (HBx) and L-HDAg can synergistically activate the serum-response element (SRE)-dependent pathway [60]. Thus, these studies highlight the differences in intracellular signal transduction induced by the small and large HDAg, and their distinct roles, despite their structural similarities. Further studies are necessary to dissect the functions of these two isoforms in the pathogenesis of HDV-induced liver disease.

Silencing of tumor-suppressor genes through aberrant DNA methylation is a frequent epigenetic event in HCC [61,62]. While DNA methyltransferase-1 (DNMT-1) is responsible for the maintenance of methylation during DNA replication, DNMT-3a and -3b catalyze de novo methylation events and are potentially oncogenic [63]. The ability of HDV to interfere with DNA methylation has been investigated in some studies. Using hepatoma cell lines, Benegiamo et al. [64] showed that L-HDAg induces DNMT-3b overexpression through STAT-3 activation. They also found that the E2F1 promoter, which is a cell cycle regulator [65], was hypermethylated in Huh7 cells expressing HDV antigens [64]. In addition, HDV was shown to induce cell cycle disruption and G2/M phase arrest [64].

Another protein that has been involved in hepatocarcinogenesis is clusterin, which was found to be overexpressed in various human malignancies [66]. Using a hepatoma cell line (Huh7), Liao et al. [67] demonstrated that both isoforms of HDAg enhance the expression of clusterin via histone H3 acetylation within the clusterin promoter [67], providing additional evidence that HDV may exert a role in hepatocarcinogenesis. The expression of clusterin was found to increase substantially in metastatic HCC compared with primary tumors [68]. HDV can also induce liver injury and hepatocarcinogenesis through the downregulation of glutathione S-transferase p1 (GSTP1), a gene that plays a critical role in the detoxification and reduction of ROS [69,70]. Inhibition of GSTP1 expression is a mechanism that has been extensively investigated in HCC tumorigenesis [69]. GSPT1 is abundantly expressed in normal human tissues, including the liver, but is significantly downregulated in most HCC [69]. By transfecting a human fetal hepatocyte cell line with S-HDAg, Chen et al. [70] demonstrated that S-HDAg inhibits GSTP1 expression by binding directly to GSTP1 mRNA, leading to the accumulation of ROS and to increased cellular apoptosis, a mechanism that may promote hepatocarcinogenesis.

## 7. Molecular Profile of HDV HCC

**Figure 1:**
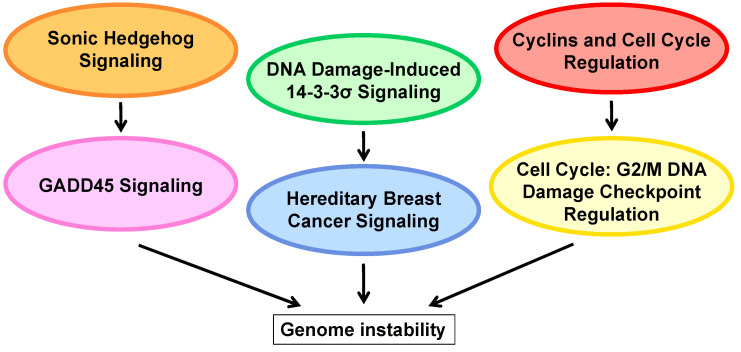
HDV-associated HCC has a unique molecular signature characterized by an enrichment of upregulated genes involved in cell-cycle/DNA replication, and DNA damage and repair, pointing to genome instability as an important mechanism of HDV hepatocarcinogenesis.

Taking advantage of a unique collection of liver specimens from patients with HDV-associated HCC and non-HCC HDV cirrhosis who underwent liver transplantation for HCC or end-stage liver disease, Diaz et al. [42] provided the first molecular profiling of this tumor. Since the liver contains a heterogeneous population comprising hepatocytes and nonparenchymal cells, gene expression profiling was performed using laser capture-microdissected malignant and non-malignant hepatocytes, tumorous and non-tumorous liver tissue from patients with HDV-HCC, and liver tissue from patients with non-HCC HDV cirrhosis. These analyses identified 547 genes and 385 genes differentially expressed in microdissected malignant hepatocytes and in tumor from whole liver tissue, respectively. About 50% of these genes were in common, indicating that a significant proportion of genes expressed by malignant hepatocytes may also be identified in whole liver tissue, despite the presence of a heterogeneous cell population within the liver tissue [42]. Using both liver tissue (77%) and microdissected hepatocytes (68%), the vast majority of genes were downregulated. Transcriptomics of malignant hepatocytes identified 20 top-scored pathways. The most significant pathways include hepatic fibrosis and hepatic stellate cell activation with all genes downregulated, suggesting that the production of extracellular matrix was indeed inhibited within the tumor; the second pathway was STAT3, which plays a major role in normal development, as well as in the regulation of cancer metastasis [71]. Interestingly, several human cancers were found to contain constitutively activated STAT-3 protein [55]. Other pathways include a few metabolism-associated pathways comprising amino acid metabolism (histidine degradation), lipid metabolism/signaling (eicosanoid signaling), hormone metabolism (estrogen biosynthesis) and vitamin metabolism (pyridoxal 5′-phosphate salvage pathway). However, the most important finding in HDV-HCC was represented by a group of six pathways (Sonic Hedgehog signaling, GADD45, DNA damage-induced 14-3-3σ, cyclins and cell cycle regulation, cell cycle: G2/M DNA damage checkpoint regulation, and hereditary breast cancer), with the vast majority (>80%) of upregulated genes involved in several interrelated functions inherent to cell/cycle DNA replication, DNA damage and repair [42] (Figure 1). The finding that the vast majority of genes were upregulated in this group of six pathways is significant, considering that HCC-HDV, as well as HBV-and HCV-associated HCC [36,43], is a disease characterized by a marked gene downregulation. The molecular signature of HDV-HCC was associated with an enrichment of upregulated genes involved in cell-cycle/DNA replication, DNA damage and repair, pointing to genome instability as an important mechanism in HDV-induced hepatocarcinogenesis (Figure 1). These genes modulate the mechanisms that recognize DNA damage, prevent cells with damaged DNA from entering mitosis or promote DNA repair [42].

Since HDV is a defective virus that always coexists with HBV, Diaz et al. [42] compared the molecular pathways identified in HDV-HCC with those of malignant hepatocytes derived from patients with HBV alone (Table 1). Of the six pathways involved in cell cycle/DNA replication, DNA damage and repair, detected in HDV HCC, none were detected in malignant HBV hepatocytes. Thus, despite the dependence of HDV on HBV, these findings suggest that the molecular signature of HDV-HCC is markedly different from that of HBV-HCC. The molecular pathways of malignant HBV hepatocytes were primarily associated with metabolic processes, retinoic acid receptor, cell remodeling, and motility functions [36]. Thus, at variance with HBV-associated HCC, in which the hallmark was a metabolism switch-off, the HDV gene signature points to genetic instability as an important mechanism of HDV hepatocarcinogenesis, suggesting that HDV and HBV promote carcinogenesis by distinct molecular mechanisms [36,42].

The genomic landscape of HCC, including HDV-HCC, was recently described in a study conducted in Mongolia [72], which has the highest incidence of HCC in the world. In this country, HCC is the most prevalent tumor, accounting for about 40% of all cancers [73]. Chronic hepatitis B and C are responsible for over 90% of all HCC cases [74], but HDV coinfection is also highly endemic, being present in 60% of the HBsAg-positive population [75]. The genomic landscape of Mongolian HCC, derived from 76 patients with HCC-associated with HBV, HCV, and HDV [72], showed previously known common driver mutations, such as TP53 and CTNNB1, but also a series of unique driver genes, most notably GTF2IRD2B, PNRC2 and SPTA1, that were not previously reported, as well as complex mutation signatures linked to Mongolian liver tumors. Interestingly, the driver gene SPTA1 was associated with HDV infection. These data suggest the existence of novel molecular mechanisms that may play a role in hepatocarcinogenesis in Mongolia [72]. Further studies on larger cohorts of patients will be important to investigate the functional role of these newly described driver genes.

## 8. Conclusions

Although HDV has not yet been included in the list of carcinogenic viruses, the evidence so far accumulated suggests that the risk is higher in patients with chronic hepatitis D compared to those monoinfected with HBV. HDV replicates in the nucleus of hepatocytes, and, through its ability to interact with several cellular proteins and to modulate their expression, it may alter multiple cellular signaling pathways involved in inflammation, oxidative stress, apoptosis, and cellular proliferation. Although many studies have been performed in vitro, there is very limited information on the molecular mechanisms of HDV-associated carcinogenesis in humans. The first molecular profile of this tumor was recently reported in a study conducted in Caucasian patients with HDV-HCC, in which gene expression was performed in microdissected malignant and nonmalignant hepatocytes [42]. HCC associated with HDV was shown to be characterized by the upregulation of genes involved in the control of cell/cycle/DNA replication, and DNA damage and repair, pointing to genome instability as an important mechanism of hepatocarcinogenesis. This genomic profile was peculiar to HDV and distinct from that of HBV-associated HCC, suggesting that these two viruses promote hepatocarcinogenesis by different mechanisms. Another recent study, which described the genomic landscape in Mongolia [72], has added more information on the molecular characteristics of HDV-HCC. However, the fact that these studies are so few and limited in size highlights the need to conduct multicenter studies in order to gather more robust data. This would be beneficial not only to better understand the role of HDV in carcinogenesis, but also to identify differences and similarities among the different forms of viral-associated HCC, which still represents one of the major challenges for the scientific community.

## Figures and Tables

**Table 1:** Comparison of the top-ranked canonical pathways of genes differentially expressed in malignant hepatocytes (MH) using laser-capture microdissection (LCM) between HDV-HCC and HBV-HCC. For each pathway, -log(p) is the statistical significance expressed as the negative log of the P value of Fisher exact test; down (%) and up (%) are the percent ratio between the number of downregulated and upregulated genes present in the dataset and the total number of genes present in the database of Ingenuity Canonical Pathways [42]. The red boxes indicate the four common pathways between HDV-HCC and HBV-HCC.

HDV LCM MH Genes	-log (p)	Down (%)	Up (%)	HBV LCM MH Genes	-log (p)	Down (%)	Up (%)
Hepatic Fibrosis/Hepatic Stellate Cell Activation	3.8	7.6	0	LPS/IL-1 Mediated Inhibition of RXR Function	7.8	13.2	2.7
STAT3 Pathway	3.3	8.2	2.7	PXR/RXR Activation	7.8	26.8	0
Histidine Degradation III	3.1	25	12.5	Bupropion Degradation	7.5	43.9	0
Sonic Hedgehog Signaling	3.1	6.7	10	Acetone Degradation I (to Methylglyoxal)	7.3	42.2	0
Eicosanoid Signaling	3	9.4	1.6	Estrogen Biosynthesis	6.4	29.6	2.7
GADD45 Signaling	3	0	21.1	FXR/RXR Activation	6.4	17.3	0.8
DNA Damage- Induced 14-3-3σ Signaling	3	0	21.1	PPARα/RXRα Activation	5.7	9.5	5.6
Estrogen Biosynthesis	2.7	10.8	2.7	Remodeling of Epithelial Adherens Junctions	4.8	2.9	17.6
Complement System	2.7	13.5	0	Growth Hormone Signaling	4.7	17.3	2.9
Cyclins and Cell Cycle Regulation	2.5	1.3	7.7	Germ Cell-Sertoli Cell Junction Signaling	4.6	5	9.4
Atherosclerosis Signaling	2.4	7.3	0	Nicotine Degradation III	4.5	20.3	1.9
Pyridoxal 5’-phosphate Salvage Pathway	2.3	3.1	6.2	Melatonin Degradation I	4.3	19.2	1.8
Extrinsic Prothrombin Activation Pathway	2.2	18.8	0	Acute Phase Response Signaling	4.2	10.6	2.9
Cell Cycle: G2/M DNA Damage Checkpoint Regulation	2.1	0	10.2	IGF-1 Signaling	4.1	11.3	5.1
Growth Hormone Signaling	2.1	7.3	1.4	LXR/RXR Activation	3.9	13.2	1.6
Coagulation System	2	11.4	0	Superpathway of Melatonin Degradation	3.9	17.7	1.6
LXR/RXR Activation	1.9	6.6	0	Complement System	3.8	21.6	2.7
Glioma Invasiveness Signaling	1.9	7	1.8	Nicotine Degradation II	3.8	17.4	1.6
Production of NO and ROS in Macrophages	1.8	5	0.6	Fatty Acid β-oxidation I	3.8	19.9	6.7
Hereditary Breast Cancer Signaling	1.8	0.8	5.4	Rac Signaling	3.7	5.8	9.6
